# Evolutionary history of bacteriophages with double-stranded DNA genomes

**DOI:** 10.1186/1745-6150-2-36

**Published:** 2007-12-06

**Authors:** Galina Glazko, Vladimir Makarenkov, Jing Liu, Arcady Mushegian

**Affiliations:** 1 Stowers Institute for Medical Research, 1000 E 50th St., Kansas City, MO 64110, USA; 2 Department of Biostatistics and Computational Biology, University of Rochester Medical Center, Rochester, NY 14642, USA; 3 Departement d'informatique, Université du Québec à Montreal, C.P.8888, suc.Centre-Ville, Montreal, QC, H3C 3P8, Canada; 4 Interdisciplinary Graduate Program in Biomedical Sciences, Kansas University Medical Center, Kansas City, KS 66160, USA; 5 Department of Microbiology, Molecular Genetics, and Immunology, University of Kansas Medical Center, Kansas City, KS 66160, USA

## Abstract

**Background:**

Reconstruction of evolutionary history of bacteriophages is a difficult problem because of fast sequence drift and lack of omnipresent genes in phage genomes. Moreover, losses and recombinational exchanges of genes are so pervasive in phages that the plausibility of phylogenetic inference in phage kingdom has been questioned.

**Results:**

We compiled the profiles of presence and absence of 803 orthologous genes in 158 completely sequenced phages with double-stranded DNA genomes and used these gene content vectors to infer the evolutionary history of phages. There were 18 well-supported clades, mostly corresponding to accepted genera, but in some cases appearing to define new taxonomic groups. Conflicts between this phylogeny and trees constructed from sequence alignments of phage proteins were exploited to infer 294 specific acts of intergenome gene transfer.

**Conclusion:**

A notoriously reticulate evolutionary history of fast-evolving phages can be reconstructed in considerable detail by quantitative comparative genomics.

**Open peer review:**

This article was reviewed by Eugene Koonin, Nicholas Galtier and Martijn Huynen.

## Background

The interest in bacteriophage biology may be at its all-time high, with the new appreciation of phage ubiquity, improved understanding of the role played by phages in controlling host abundance and in host genome evolution, and because phage genomes are the source of useful molecular reagents and new antibacterial compounds. The ongoing sequencing of phage genomes produces the unprecedented amount of data, which is indispensable for understanding phage biology.

In spite of these advances, the taxonomic diversity of phages is just beginning to be assessed. The International Committee on the Taxonomy of Viruses (ICTV) recognizes several groups of phages, on the basis of shared morphological traits, such as the shape, size, and structure of the virions, with some consideration of other molecular properties, such as the structure of genomic nucleic acids and similarities in the signature genes [1]. For example, tailed bacteriophages with double-stranded DNA genomes attain the rank of the order *Caudovirales *and are divided into three main families according to their tail morphology: *Siphoviridae *(long noncontractile tail), *Myoviridae *(long contractile tail), and *Podoviridae *(short tail). Within these families, genera are defined by other molecular traits, such as the *cos *or *pac *sites, terminal redundancy and circular permutation of the genome, concatemer formation, modified bases, and the presence of DNA polymerase or RNA polymerase genes. While these families account for more than 60% of *Caudovirales*, about one-third of all tailed phages remain classified no further than the family level. The proportion of unclassified phages is comparably high in almost all phage groups. Moreover, evolutionary relationships between phages with different particle morphology are not well studied. With hundreds of bacteriophage genomes completely sequenced, it is now appropriate to undertake the systematic inventory of molecular characters and to see whether it provides us with better understanding of phage evolution and with means to refine phage taxonomy.

Phages with dsDNA, the subject of this study, account for about 65 percent of all phages with completely sequenced genomes, and infect hosts from various clades of Bacteria and Archaea. No genes are shared by all dsDNA phages, or even by a majority of them [2], so the evolutionary reconstructions and taxonomic proposals for this group cannot be based on the analysis of sequence alignments, as it has been possible for cellular organisms, which all have homologous rRNA and dozens of other universally conserved genes [3-6]. In 2002, Rohwer and Edwards [7] proposed a way to get around this limitation by using information about shared homologous genes as a measure of similarity between phages: the pairwise distances between genomes (which, in their case, combined the counts of shared genes with the degree of sequence similarity between matched pair of genes) can be used for phylogenetic inference with distance matrix-based approaches. The study of Rohwer and Edwards confirmed the existence of several phage groups previously suggested on the basis of morphology or single-gene phylogenies, but the monophyly of the largest groups of DNA-containing bacteriophages, such as the family *Siphoviridae*, was not supported in their "proteomic tree" [7].

In an earlier work, we have reviewed the approaches to comparative genomics and taxonomy of dsDNA phages and delineated Phage Orthologous Groups (POGs), the sets of orthologous genes that are shared by three or more phages each [2]. The sensitive algorithm of POGs construction, which does not rely on the arbitrary score cut-off [8], may be especially suitable for viruses, which appear to have high percentage of short ORFs and fast-evolving gene products. The coverage of phage genomes by POGs is on average 52 percent, and it remains quite high – 42 percent – even for the unclassified phages, suggesting dozens of characters suitable for phylogenetic inference in most phages.

In the same study, we introduced 'phageness', i.e., the specific association of a gene with phage or prophage genomes, as opposed to the host genomes [2]. Determination of phageness of each POG indicates that about 80 percent of POGs are rarely or never exchanged with the host genomes, and further 8 percent of POGs appear to be monophyletic within phages, most likely having been gained by the phage kingdom in a single transfer event [2]. Thus, even though many host genes have been accrued by the phage kingdom as a whole, the vast majority of POGs are phylogenetic characters that are robust against repeated phage-host gene exchange, and may be useful phylogenetic markers. On the other hand, phage-specific genes are transferred within the phage kingdom itself, producing a reticulated evolutionary history of phage genomes, i.e., a phylogeny that has extra edges between some branches and is no longer a 'tree' in a formal, graph-theoretical sense. This type of horizontal gene transfer (HGT) has been postulated as a major factor in bacteriophage evolution more than 25 years ago by David Botstein [9], who emphasized homologous recombination between DNA genomes of co-infecting phages as the main mechanism of HGT. More recently, numerous examples of mosaicism in phage genomes, best explained by gene exchanges in the absence of homologous DNA sites, have been documented (reviewed in [10-12]). Any realistic reconstruction of phage evolutionary history needs to take into account HGT, and in this study, we use the information about gene content in phages with dsDNA genomes, in conjunction with phylogenetic signal contained in the alignments of phage-encoded proteins, to infer such a reticulated evolutionary history.

## Results and Discussion

The set of dsDNA phages used in this study comprises all complete genome sequences deposited in GenBank before 2005 (Table S1, available as Additional File 1). These genomes contain conserved genes that belong of 981 POGs, including 803 POGs that appear almost never to be exchanged with the host genomes [2]. These 803 POGs in 158 genomes are the main character set that we used to infer the gene-content tree of bacteriophages.

At the first step toward understanding the evolutionary history of phages, we built a conventional phylogenetic tree on the basis of gene content. The tree contains only bifurcations and no reticulations, even though, as discussed above, our dataset also contains information about (possibly frequent) gene exchanges between phages. We reasoned that, although HGT will be initially ignored, we might still be able to discover robustly supported, evolutionarily informative partial phylogenies, in the cases when vertical gene inheritance is the dominant mode of evolution in a particular phage lineage. We can then augment this initial picture by inferring reticulation events.

**Figure 1:**
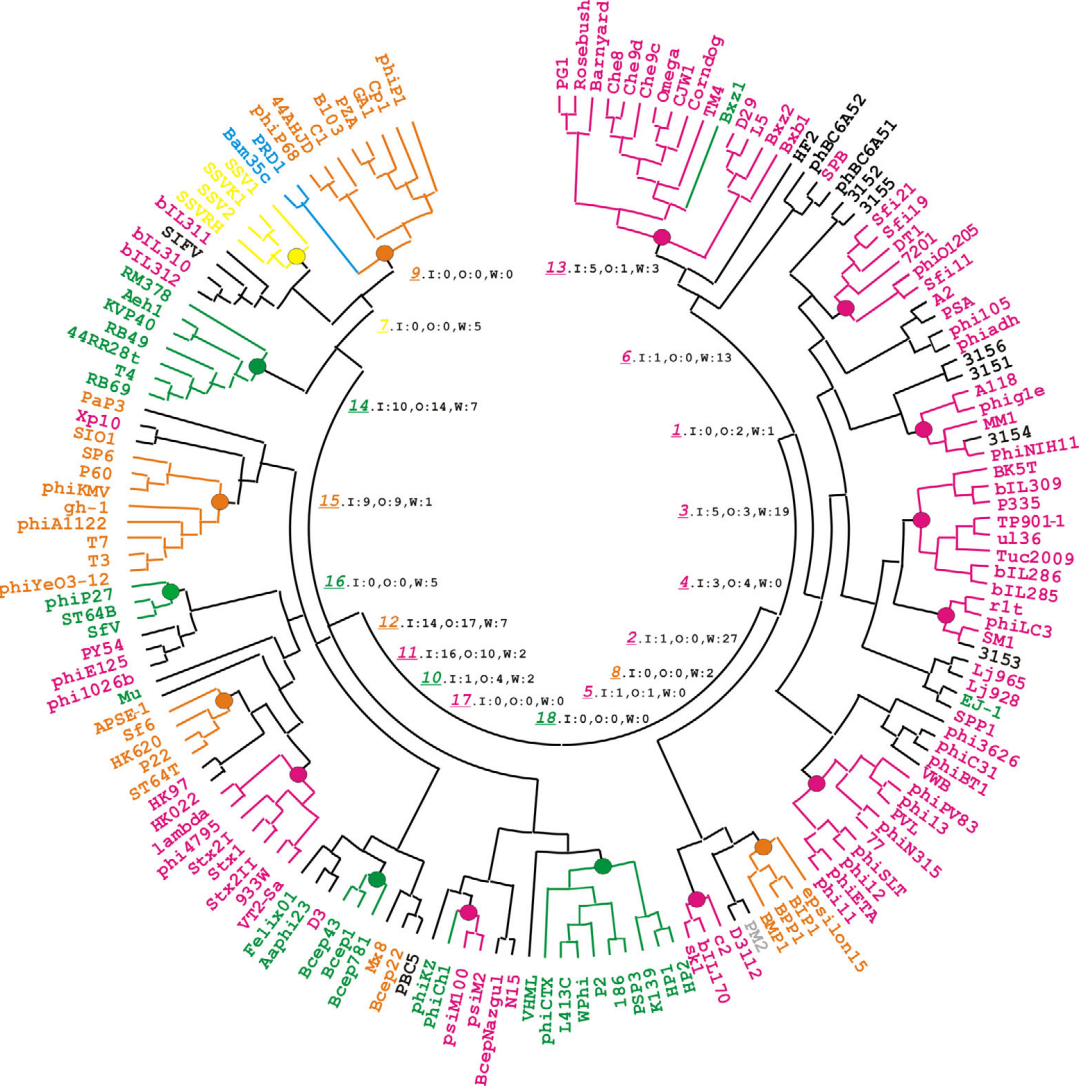
**Bacteriophage phylogeny inferred from gene content**. The tree was built using generalized average distance and neighbor-joining algorithm (see Methods). Large dots indicate clades inferred in the majority of resampled data sets. The branches leading to individual phages are colored according to their ICTV classification: family *Siphoviridae *is in magenta, *Podoviridae *is in orange, *Myoviridae *is in green, *Fuselloviridae *is in yellow, and *Tectiviridae *is in blue. The Bayesian tree displaying essentially the same phylogeny is presented in Fig. S1, available as Additional File 1. In the inner circle, the italicized, underlined colored numbers indicate 18 well-supported phage groups (see Supplementary Text for groups' description). They are followed by summary information of horizontal transfer events, where I stands transfer into the group, O for transfer from this group to another group, and W for transfer within the group. The algorithm for reconstructing these events is described in Methods and illustrated in Figure 2.

The symmetric matrix of all pairwise distances between phage gene content vectors (see Methods) was used to build tree by the neighbor-joining algorithm. In a fundamentally different approach, we also constructed tree directly from the POG presence-absence characters using Bayesian inference. In addition, we prepared datasets in which the presences and absences of each POG across the genomes were jumbled, and built trees from these datasets for comparison with the real data. Considerable phylogenetic signal was captured by both distance matrix-based and Bayesian trees when real data were analyzed (Figure 1 and Figure S1 and Table S1, available as Additional Files 2 and 1, respectively). In total, 112 phages, or 71 percent of all studied genomes, were classified into 18 groups, which enjoyed moderate to strong statistical support, 53 % on the average. In contrast, analysis of trees obtained from the jumbled data matrices revealed no strongly supported clades (average support of 12.8%, s.d. = 0.23; see Additional File 3 for statistical details).

The groups indicated in Figure 1 contain between 3 and 15 phages, with the average clade size of 6 genomes. The biological plausibility of these groups is underscored by the fact that each of them includes at least some phages that are recognized as the nearest relatives in the ICTV-approved taxonomy or on Rohwer-Edwards' "proteomic tree" [7]. Closer inspection, however, indicates that there are three distinct ways in which our phage groups relate to the ICTV taxa: *i*. groups including phages from only one ICTV-approved genus (6 such groups with 39 phages), *ii*. groups corresponding to an ICTV-approved genus but containing additional species from the same family (7 groups, 36 phages), and *iii*. groups containing species from different ICTV families and sometimes also from "unclassified bacteriophages" (5 groups, 37 phages). All three categories are supported by comparable average number of shared POGs.

The first category comprises groups that include only phages from the one and the same ICTV-approved genus. These are Groups 7, 11, 12, 14, 15 and 18 (Table S1 in Additional File 1). Group 7 consists of *Sulfolobus *spindle-shaped viruses 1, 2, Kamchatka-1 and Ragged Hills, coinciding with family *Fuselloviridae*. Coliphage lambda joins five Shiga toxin-converting phages in Group 11. Group 12 consists of three short-tailed P22-like phages Sf6, HK620, and ST64T, as well as the *Enterobacteriaceae *phage P22 itself. Group 14 includes T-even phages RB69 and T4, pseudoT-even phages RB49 and 44RR2.8t, and SchizT-even phages Aeh1 and KVP40, as well as RM378 T4-like phage that infects the thermophilic bacterium *Rhodothermus marinus*. Members of Group 15 are included in ICTV-approved T7-like genus, except for an outlier, *Synechococcus *phage P60, which currently is classified as unassigned member of *Podoviridae *by ICTV. Phages in Group 18 are all P2-like viruses, sharing common morphology and several other traits.

Each group in the second category corresponds to an ICTV-approved genus but contains several more species from the same family. These are Groups 2, 3, 6, 5, 8, 10, and 16, tentatively classified at the family level as siphoviruses (Groups 2, 3, 6, 5), podoviruses (Group 8) or myoviruses (Groups 10 and 16). Nine phages are included in Group 2, all of them temperate phages or prophages from *Staphylococcus aureus*. Evolutionary closeness of phages in this group is manifest at the levels of nucleotide sequence, protein sequence and genomic organization [13]. Group 3 and Group 6 consist of phages of Gram-positive dairy bacteria *Lactococcus lactis *and *Streptococcus thermophilus*, respectively. Both groups include phages sharing significant sequence similarity throughout the genome [11]. Group 5 includes three *Lactococcus lactis *phages that are siphoviruses with high sequence identity [11]. Group 8 consists of four podoviruses – three closely-related *Bordetella *phages and *Salmonella *phage ε 15. Group 10 includes three unclassified myoviruses infecting *Burkholderia cenocepacia*. Group 16 consists of coliphage P27, *Salmonella *phage ST64B, and *Shigella *phage SfV, all believed to be the lambdoid/Mu chimeras [14].

The third category, including groups 1, 4, 9, 13, and 17, is perhaps the most interesting. The phages in Group 1 infect *Bacilli*. Three of them are siphoviruses, the fourth, phage 315.4, is currently unclassified by NCBI. Similarly, Group 4 contains three siphoviruses – two infecting *Lactococcus *and one infecting *Streptococcus *– as well as an unclassified bacteriophage 315.3. Group 9 contains eight podoviruses and two phages belonging to the family *Tectiviridae *– the enteric PRD1 phage and *Bacillus thuringiensis *phage Bam35c. The phylogenetic affiliation between the two morphologically distinct groups of phages, tectiviruses and PZA-like podoviruses, has been also noted by Rohwer and Edwards [7]. We note that this group is defined by a single shared character, albeit an important one, a protein-primed DNA polymerase (POG52). Fifteen phages isolated from *Mycobacterium *species form a monophyletic Group 13: all of them have siphovirus-type morphology, except for *Mycobacterium *phage Bxz1 that possesses a myovirus-like contractile tail. This group is also defined by a single shared hypothetical protein (POG921). Rather than dismissing these two groups as spurious, we consider them plausible working hypotheses, which may be further evaluated by including new characters into analysis or by more sensitive detection of remote similarities between gene sequences. Group 17 consists of three tailed phages infecting archaeal hosts; two of them, psiM2 and psiM100, have siphovirus-like morphology, whereas phiCh1 has a myovirus-like contractile tail.

Phages in most groups tend to have overlapping host ranges, at least down to the bacterial family level. This may be indicative of both vertical inheritance of the core set of genes in these groups and horizontal gene transfer between phages that can co-infect the same hosts. These possibilities are analyzed in more detail below.

Forty-six phages remained singletons because of insufficient statistical support for their inclusion into any group. Nearly one-half of these phages are unclassified siphoviruses, and the rest is almost equally distributed between unclassified podoviruses, myoviruses and 'unclassified bacteriophages'. This, as well as the fact that none of the ICTV families resolved as a single robustly supported clade, indicates that phages with similar particle morphology do not always share a set of recognizably homologous genes sufficient for reliable placement of these phages in the phylogeny. Further analysis, including searches of updated databases, more sensitive ortholog definition, and perhaps inclusion of additional molecular and morphological characters, may help to place many of these singletons on the tree, as well as to lend better statistical support to the internal branches.

All told, our trees recovered considerable phylogenetic signal, with very few differences between the neighbour-joining and Bayesian tree. The case of placement of BcepNazgul phage is illustrative of these occasional differences. Phages BcepNazgul and N15 share POG852, POG853, POG967, and the same three POGs are shared by BcepNazgul and lambda phage. The two approaches break the ties differently. In the Bayesian tree, N15 and PY54 are grouped together, but they are also grouped with VHML, which shares only 1 POG with N15 and PY54. In the NJ tree, PY54 is grouped together with phiE125 and phi1026b6, sharing 15 POGs. On balance, the NJ tree appears to give biologically more plausible solution.

There is no doubt that hierarchical representation is not sufficient to provide a complete picture of phage evolution, for the already mentioned reason of HGT that is thought to be frequent in phages. HGT has been also recognized as a confounding factor in the attempts to reconstruct the evolutionary history of cellular prokaryotes, and representations of evolutionary history that allow reticulations have been advocated for bacteria and archaea [15-18]. Several algorithmic and statistical approaches to address this problem have been proposed (e.g., references [16] and [19-22]), but much remains to be done, especially given that none of the existing methods is equipped to specifically address the challenges of virus genomics discussed above. To address this problem in a novel way, we used new modification of the T-REX algorithm (see Methods) for automated detection of HGT events.

**Figure 2:**
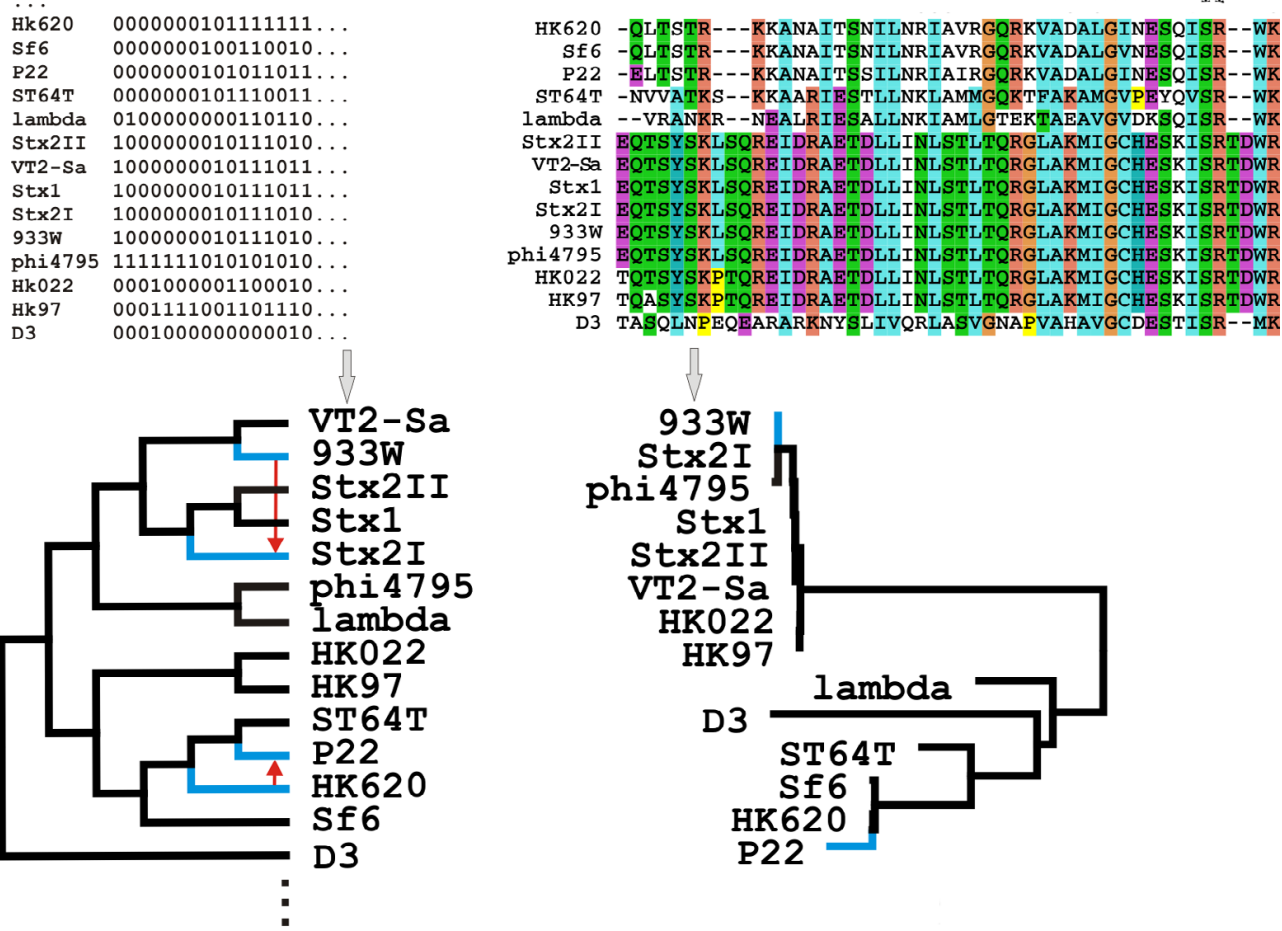
**Inference of horizontal gene transfer between phages**. Phage genome tree is inferred from gene content data (left side of the top panel) and sequence family trees are inferred from the aligned sequences, separately for each POG (right side of the top panel). The T-REX algorithm is used to infer HGT events by choosing such rearrangements of the gene content tree that reattach the subtrees in a way that minimizes the Robinson and Foulds topological distance to the appropriate sequence family tree. On the top right, a fragment of sequence alignment for one class of cII transcription regulators (POG226) is shown. The sequence family tree built on the basis of complete alignment is shown at the bottom right corner, and the sub-tree of the gene-content tree that contain the same set of phages as thesequence family tree is shown at the bottom left corner. Two pairs of phages, namely, 933W and Stx2I, as well as HK620 and P22, are in discordant positions in the gene content and protein family trees (indicated by the blue edges in both trees). To reconcile gene content and protein family trees, T-REX suggests a transfer from 933W to Stx2I and from HK620 to P22 (blue arrows).

The essence of the T-REX approach is in comparison of two trees: a larger gene content tree *T*, such as the one shown in Figure 1, and a smaller tree constructed on the basis of the alignments of protein sequences from each individual POG (a sequence family tree *T*_*sf*_). The number of sequence family trees is in principle the same as the number of POGs, though, for algorithmic reasons, only POGs with four or more members are used. Each *T*_*sf *_contains at its tips only a subset of phages that are included in *T*, because most genes are found in a small number of phages. The gene-content tree can be pruned to retain only those tips that are also present in the sequence-family tree, and such pruned tree *T*_*gc *_exists for each *T*_*sf*_. The topology of *T*_*sf *_is compared with that of *T*_*gc*_, and the incongruence in two tree topologies is interpreted as the evidence of recombination/reticulation events that can be inferred using the specific constrained optimization criteria (See Methods for details and Fig. 2 for an example).

The analysis of incongruencies between gene content tree and sequence family trees using T-REX algorithm revealed 294 putative acts of gene transfer (Table S2, available as Additional File 1), which involved 114 of 158 phage genomes and 229 POGs. Despite these large absolute numbers, the results mean that significant fraction of phage genomes, and, notably, the majority of POGs have not been involved even in a single phage-phage HGT event.

"Promiscuous" phages are relatively rare: only 11 percent of all phages appear to have acquired five or more genes in the past, and only 10 percent have donated five or more genes (Fig. S2 and Table S3, available as Additional Files 4 and 1, respectively). "Vagabond" POGs are even rarer: 42 POGs appear to have been transferred twice, seven POGs were transferred three times, and only three POGs were transferred four times. These repeatedly transferred genes encode structural proteins, enzymes, transcription factors, and uncharacterized proteins, in almost the same proportion as within POGs in general (Table S4, available as Additional File 1). Thus, even though some of functional modules in phage genomes, such as lysis cassettes, could be viewed as particularly autonomous and suitable for recombination with different sets of replication and transcription factors, or of capsid proteins, it appears that HGT does not strongly favor genes with any specific molecular function. On a more general note, the trends of HGT distribution in phage genomes and in POGs that we observed appear to be in agreement with our earlier results on phage-host gene transfer [2] and with the same trend in gene families and in several groups of Bacteria and Archaea [20,23], in that such distributions tend to be fat-tailed: that is, most genes in most genomes have been horizontally transferred only rarely, but a smaller proportion of genes have been transferred many times. These processes result in a large absolute number of HGT and simultaneously in a large proportion of gene families that are free of HGT.

The HGT between phages is more common within groups, with only 4 groups with repeated between-group transfers detected in our analysis (Figure 1 and Table S3). Interestingly, the phages most active as gene donors and those most active as gene recipients are not always the same, with only 7 phages seen in both these categories. The leaders in a number of transfers in both directions are Group 14, which consists of T4-like myoviruses with large genomes, and Group 12, consisting of P22-like podoviruses.

The observation of the high rate of the within-group gene transfer and low rate of between-group transfer begs the question of whether the phage groups in the gene-content trees are in the first place defined by vertical inheritance, or are the artifacts of HGT. To distinguish between these two possibilities, we excluded from our dataset all POGs which were transferred at least once (229 POGs) and used the remaining 594 POGs to infer the genome tree again. This step did not lead to radical changes in the clades of the tree, but two types of differences were observed. First, a minority of the phage groups described earlier (four groups out of eighteen) lost one or two members. Second, the average statistical support for 18 groups decreased somewhat, from 73.5% to still considerable 63.4%, with the bootstrap values falling below 30% in four groups and actually increasing in three of them. There was no strong correlation between the change in support and either the frequency of the HGT events involving the members of the group or the ICTV approval status of the group (Table S5, available as Additional File 1). These observations indicate that the inferred HGT events, frequent as they may be in the absolute terms, are nevertheless not so frequent as to obscure the pattern of vertical, divergent evolution of phage genomes, at least among the groups defined in this study.

The plausibility of reconstructing evolutionary history and of building evolutionary classification of bacteriophages has been drawn into question, and it has been proposed to retain hierarchical structure at the higher levels of phage classification, where "domains" correspond to distinct lineages of phages with different forms of genetic material, and "divisions" group together those phages that exhibit little or no evidence for genetic exchange with other divisions. At a more shallow level, it has been proposed to establish various "modi", and a phage to simultaneously belong to more than one modus [26]. This framework explicitly recognized HGT and the ensuing reticulate relationships between phages, but it did not offer the consistent way of deriving the modi in the first place, and did not answer the evolutionary question about the set of past events explaining the makeup of the observed phage genomes. In the current study, we proposed the algorithmic approach that is suitable for answering these questions. At the same time, we defer the issues of phage nomenclature and taxonomy to a later time – essential as these questions are, they may be better addressed after we define the evolutionary relationships and natural groups that have to be properly named.

We used patterns of gene content conservation in phage genomes to investigate their evolutionary history, by first constraining the relationship graph to a tree-like topology and detecting well-supported groups of phages, which come close to the ICTV phage taxonomy at the genus level, and then augmenting this inference with analysis of several hundred of the sequence-based trees of individual gene families. In contrast to the study of Rohwer and Edwards, which used gene content to infer reticulation-free phylogeny [7], our approach is to exploit the discordance between the topologies of phage genome trees and protein family trees and to infer the recombination events between phage lineages on the basis of this discordance. The main shortcoming of our approach is that it does not resolve the deep clades of phage phylogeny – the problem that is also encountered in phylogenetic studies of the anciently divergent cellular organisms [5,6].

A note of caution is also due with regards to the HGT rates determined by T-REX. Our attempt at quantification of the HGT events may suffer from several confounding problems, some of which lead to overestimation, and other to underestimation of the HGT rate. The former type of problems may have to do with statistical errors in tree inference, when difference between tree topologies is interpreted as HGT, even though one or both nodes in question have insufficient statistical support (note that, on the other hand, comparing only well-supported nodes may result in underestimated HGT rate). Overestimation of HGT is also likely to occur as the size of subtrees under comparison grows, as there may be more low-cost HGT scenarios in small trees than in large ones (see reference 27 for a recent discussion of related algorithmic issues). We feel, however, that these effects may be overwhelmed by the opposite trends that lead to underestimation of the number of HGT events. Indeed, an ORF has to be found in more than three genomes in order for our method to work in the first place, which removes from consideration small POGs. Moreover, the phylogenetic signal may be low in the gene-content subtrees because of insufficient number of characters and parallel loss of characters, and in sequence family trees because of rapid sequence evolution and/or mutational saturation. Finally, gene exchanges between two nearest neighbors in the tree are ignored by all existing methods of HGT inference [28].

The observation of the one-tailed distributions of HGT events in phage genomes and in POGs appears to agree with the data on genomes of cellular prokaryotes [20,23,24] as well as more qualitative observations for different subsets of bacteriophages [25,29]. It means that most genes have been horizontally transferred either once or never, and only a relatively small proportion of genes have been transferred many times. Note that the latter small proportion nevertheless in itself may be a large number, if the total number of genes in the sample is itself large, as is the case here and everywhere in comparative genomics. Though different techniques of HGT detection may give different estimates of the absolute number of HGTs, we there have not been any evidence for other types of distribution for viral or bacterial HGTs in the literature (e.g., that it is uniform, or that it has a theoretical, as opposed to sample, mean). We believe that these observations can help us move away from the extreme views on HGT role in the evolution of life and to bring about a more balanced view, in which large absolute number of genes horizontally transferred at some point in their history may be high, and the proportion of gene families that show evidence of HGT may be relatively low at the same time, perhaps just enough to make phylogenetic inference worth the effort.

## Conclusion

Notwithstanding the statistical and algorithmic problems, the observed cases of gene transfer between phages do not amount to the erasure of the historic record: considerable signal of vertical inheritance can be still recovered by analysis of appropriately chosen molecular characters. As of this writing, the genomes of more than 300 dsDNA phages, infecting an increasingly diverse collection of prokaryotic hosts, are available. Recent analysis of sequences accumulated in public databases has indicated that combining many molecular characters significantly improves support for nodes in large trees of cellular organisms, with good tolerance of uneven sampling and missing data [30]. We expect that this growth of phage genome databases, in conjunction with methodological improvements of HGT detection, will provide for more and more accurate reconstruction of evolutionary history of bacteriophages.

## Methods

### The POG resource

Detailed description of POG construction and analysis of the POG database has been published [2]. Here, we summarize the procedure and note some of the relevant properties of the POG resources. For each of the 164 phage genomes included in the initial analysis, we predicted ORFs de novo with the GeneMarkHMM and GeneMarkS programs [31,32], using the codon frequency model for the best-known bacterial host, and then replacing it with the codon frequency information derived from the verified ORFs. We detected 94 predicted ORFs missed by the original GeneBank submissions; for 80 of them, homologs were found in the NCBI database. The nucleotide sequence of each phage genome was also searched using the TBLASTN program [33]. We estimate that ≥ 95% of all ORFs coding for proteins larger than 5 kDa have been found [2], though the exact position of the start and stop codons in a small subset of ORFs may have be defined imprecisely (LJ and AM, unpublished observations). Multidomain proteins were split into individual domains using the HHsearch program [34]. We made all-against-all sequence comparison of the resulting mix of proteins and domains using the gapped BLASTP program [33]. Symmetric best matches, or SymBeTs, were recorded, and the COGMAKER package, which is the implementation of the original COGNITOR algorithm [35], was used to produce orthologous groups of genes by first recovering the triplets of SymBeTs in three genomes (one gene in each genome) and then merging all triangles with shared sides [8]. Unlike the NCBI COG resource, in which each SymBeTs have to occur between proteins sequences from two evolutionarily distant lineages, we treated every phage as a distinct lineage, which resulted in a moderate over-representation of small POGs and inflated POG coverage of genomes that had closely similar strains in the database [2]. The properties of these distribution tails, however, have a minor impact on the results (GG, JL, and AM, unpublished observations). We checked whether pairs of paralogous POGs would be merged if all sequences in the NCBI NR database were also considered, and found no such cases. Over one-third of the total (339 POGs) are found in 3 phage genomes. Approximately 60% of the POGs are present in 4 to 14 phage genomes, with the average number of members per POG of 6. Almost 90% of the POGs contain one representative per phage, i.e., no in-paralogs.

### Calculation of intergenome distances

Presences and absences of POGs in each phage genome were coded as binary vectors in the form **X**_**i **_= (*x*_*i*1_,.*x*_*i*2_,...,*x*_*iN*_), where *i *= 1,...,*M*, and *j *= 1,...,*N*, and *M *and *N *are, respectively, the number of POGs (*M *= 803) and the number of phage genomes (*N *= 158; the total number of phage genomes we started with was 164, but six genomes did not contain genes with high phageness value that were conserved in at least two other phage genomes). We analyzed generalized average distance measure *d*_*A*λ _with different exponents [36], as well as a measure based on Pearson correlation, to select optimal way to estimate evolutionary distances for our data set. The distance with exponent *λ *= 8 was selected on the basis of the extreme values of skewness and kurtosis: as discussed in reference 36, these values tend to be good predictors of the accuracy of the clustering solution, even though the general theory of the choice of distance measure in clustering problems is still unavailable. Several other measures, including the one based on correlation coefficient, gave nearly identical NJ trees (not shown).

### Construction of gene-content trees

The neighbor-joining trees were inferred based on the intergenomic distances *d*_*A*_∞ using the NEIGHBOR program of the PHYLIP package [37]. The statistical support for internal nodes was obtained with the delete-jackknife method [38], by randomly selecting 90% of the sample data and recalculating trees over 100 replications [39]. For Bayesian inference, we used MrBayes v.3.1.2 [40], with gamma rate distribution estimated from the data set, one cold and three incrementally heated chains run for 2,000,000 generations with random starting trees, and the temperature parameter value of 0.2. Trees were sampled every 100 generations and 10000 initial trees were burnt in.

### Construction of protein sequence family trees

We constructed alignments of sequences from 450 POGs that included protein sequences from four or more phages using the ClustalW program [41]. Four is the minimal number of leaves on the tree that can be processed by our algorithm, which requires midpoint rooting (see below). Phylogenetic trees were inferred using NJ algorithm and PC gamma distance with α = 2.25. This protein distance measure corresponds to Dayhoff model of amino acids substitutions and is very close to the popular JTT model that has PC gamma distance with α = 2.4 [42].

### Detection of horizontal gene transfers: the new version of the T-REX algorithm

For this study, we developed a new version of the T-Rex algorithm [43,44], which is described in this section and, in more detail, in Additional File 3. A protein sequence family tree *T*_*sf *_is a tree inferred from alignment of protein sequences that belong to a POG. This tree has *n *leaves that are labeled by the set of *n *bacteriophages, where *n *is much less than the total number of phages included in the analysis (the average numbers of genes in 450 POGs that contain 4 or more species is 6). We also reduce the 158-taxa gene-content tree, such as the one given in Figure 1, to a smaller tree *T*_*gc*_, containing only the leaves labeled by the set of the same *n *phages, by removing from it 158-*n *lineages corresponding to the organisms missing from the sequence family tree. Both gene-content and sequence family trees are then rooted by midpoint in order to take into account the timing constraints (See Supplementary Text). If there exist identical sub-trees with two or more leaves belonging to both *T*_*gc *_and *T*_*sf*_, we reduce the size of the problem by contracting these sub-trees, replacing them with the same auxiliary node in both *T*_*gc *_and *T*_*sf*_, and preserving this replacement throughout the computation (i.e., assuming that the branches of these sub-trees will not be involved in the HGT operations). All possible directed transformations consisting of standard Sub-tree Pruning and Regrafting (SPR), are evaluated in a way that the value of a selected optimization criterion (in our case, Robinson-Foulds distance – reference 45) between the transformed species tree and the gene tree is computed. The algorithm proceeds iteratively by testing all possible SPR operations (i.e., HGTs) between pairs of branches in the gene-content tree *T*_*k*-1 _(*T*_0_*= T*_*gc *_at iteration 1 or transformed tree at the following iterations) except the transfers between adjacent branches and those violating the constraints (see Supplementary Text). After the SPR operation is selected, the size of the problem is reduced further by contracting the newly-formed sub-tree in the transformed gene-content tree *T*_*k *_and the sequence family tree *T*_*sf*_. In the list of the obtained HGTs, the idle transfers, i.e., those whose removal does not change the topology of *T*_*k*_, are identified and eliminated. The procedure is repeated until the distance between the transformed gene-content tree *T*_*k *_and the sequence family tree *T*_*sf *_equals zero or if no more HGTs can be generated due to the violation of the timing constraints (see Supplementary Text). The computation requires *O*(*kn*^4^) operations to generate *k *transfers in a phylogenetic tree with *n *leaves. However, because of the progressive size reduction of the gene-content and sequence family trees, the practical time complexity of this algorithm is *O*(*kn*^3^).

## List of abbreviations

ICTV, International Committee for the Taxonomy of Viruses; HGT, horizontal gene transfer

## Competing interests

The author(s) declare that they have no competing interests.

## Authors' contributions

ARM, GVG and VMM designed research and wrote the manuscript; all authors performed research, analyzed the data, read and approved the final manuscript.

## Reviewers' comments

### Reviewer 1: Eugene Koonin, National Center for Biotechnology Information, National Library of Medicine, National Institutes of Health

This is an attempt on a full-scale phylogeny and phylogenetic taxonomy of dsDNA tailed phages, apparently, the largest class of "organisms" (or "biological agents" if viruses cannot be called organisms) on earth. So the importance of this paper needs no additional emphasis.

My main concern with the current version of the paper is that the gene-content tree is not really a phylogenetic tree (as pointed out in ref. 5 of this paper but more fully explicated in Wolf et al. 2002. Trends Genet.18(9):472–9). At best, it can be viewed as a phylogenetic tree in which LBA-type artifacts are inevitable and their source is obvious, i.e., species that have convergently lost similar subsets of genes will form false "clades", and to some extent, the same might happen with species that have convergently gained similar sets of genes. This problem is not at all addressed in the current version of the paper, and I think this needs to be rectified.

*Authors' response*: In gene content-based phylogenetic inference, proper normalization/weighting of the average number of shared genes appears to be an effective way of countering the "genome size attraction" artifact (for earlier discussion, see, for example, ref. 24 in this manuscript, or reviewer's own B. Mirkin and E. Koonin (2003) in M. Janowitz, J.-F. Lapointe, F. McMorris, B. Mirkin, and F. Roberts (Eds.) Bioconsensus, DIMACS Series, V. 61, Providence: AMS, 97–112). There is no general solution of the average weighting problem yet, but we think that the use of parametric family that we have proposed in ref. 36 is a sensible starting point in dealing with convergent gene losses (note that one of the examples in that study is phylogenetic inference from gene content in proteobacteria, which appears to be free of "small genome attraction" when the value of the exponent is chosen on the basis of analysis of pairwise distance distributions).

As to convergent gains of genes, we think that, in order for this process to induce the artefacts in our approach, these will have to be gains from the hosts (as phage-phage gene donations are dealt with explicitly at the second step of the analysis), and these gains will have to involve POGs with high phageness (as low-phageness POGs are not considered). These two requirements appear to work against each other. Moreover, weighting the average number of shared genes helps in this case, too.

As an aside, we feel that J.A. Lake and M.C. Rivera may have been onto something when they suggested (*Mol Biol Evol*. 2004, 21:681–690) that gene-content artifacts may be more akin to the composition effects in sequence comparison than to LBA artifacts.

The analysis of putative HGT events using updated T-Rex is, certainly, of interest and value. The results of this part are unexpected (at least, to me) in that there is rather little HGT. I am wondering whether there might be some source of systematic underestimate – perhaps, it has something to do with using the gene-content tree as the species tree?

*Authors' response*: The discussion of the HGT rates was perhaps too brief in the original version. In response to this comment, as well as the opposite concern of Nicolas Galtier (i.e., about possible overestimation of HGT rates), we expanded it, and we also discussed a broader context of these observations at the end of the revised manuscript.

The authors indicate that the detectable HGT does not amount to erasure of the vertical phylogenetic signal. I accept this for individual clades but I am wondering about the deep branches which, as the authors rightly note, are poorly resolved. Could it be that at that evolutionary depth the amount of HGT was such that it does indeed obliterate whatever vertical signal might have been there?

*Authors' response*: We agree with the reviewer that the loss of vertical signal is more likely for the deep branches of the tree. We also agree that the question should be decided on the clade-by-clade basis: if we improve our science and some deep branches become better resolved, we will care about it – even if other parts of evolutionary history continue to be unresolved (or even turn out to be non-resolvable).

I have a suggestion on a potential extra figure, one that would show the network of inferred HGT events between different phages groups.

*Authors' response*: A good way of drawing reticulated trees is still an open problem. For now, this information is present in the Additional File 1 in a tabular form.

Finally, this paper has a taxonomic component on which I do not feel competent to comment. I am sure those working directly on phage taxonomy will have something to say.

*Authors' response*: We hope so too: though none of the contacted members of the International Committee for the Taxonomy of Viruses was available for a regular review, we will try to communicate with them and seek feedback, perhaps in the form of comments at the *Biology Direct *website.

### Reviewer 2: Nicolas Galtier, CNRS-Université Montpellier II

This paper examines the gene content of 158 double-stranded DNA phage genomes in a phylogenetic way, thus defining groups of related phages. Some groups are in agreement with the existing taxonomy, some are new and provide unsuspected links between morphologically divergent phages. It is argued that horizontal gene transfers (HGT) between phages, detected by comparing individual gene trees to the gene-content tree, are not frequent enough to remove the phylogenetic signal due to vertical inheritance.

I think that clarifying the taxonomy and evolutionary mode of DNA phages is a sensible goal, and that this article addresses the problem in an appropriate way. I cannot really tell how novel this contribution is given my limited knowledge of the bibliography in this field, but as an external I liked reading the paper. I particularly appreciate the idea of detecting HGT by comparing gene trees to a gene-content tree, i.e. two independent sources of information. I have a number of technical or conceptual comments.

- I would suggest to present in this manuscript the method by which Phage Orthologous Groups (POGs) are defined. This step is critical. The reader should be able to appreciate its validity without reading the Liu et al 2006 paper.

*Authors' response*: We included a short description of the POG construction and moved much of technical information from the supplementary files into the main text.

- I was a bit surprised that only 150 genomes were analysed, when 300 are currently available. 150 is not much more than the 102 analysed by Rohwer and Edwards in 2002. "Before 2005" is an old date in the rapidly evolving field of genomics. Since one aspect of this paper is to provide a molecular taxonomic update, I think that the study would benefit from using all the available data.

*Authors' response*: There is no doubt that the more genomes the better, as mentioned in the Discussion. For various non-scientific reasons, we had to make a choice between updating the POG collection and working on phylogenetic inference, and we chose the latter. We are now working on a major update of the dataset. For the record, the number of genomes in this study is twice as large as the dsDNA subset of the genome collection used by Rohwer and Edwards (their decision to include phages with RNA genomes does not strike us as sound, but we may consider phages with ssDNA in the future analysis).

- I found the bibliography a bit lacking. In the very first paragraph, it might be useful to redirect the reader towards references illustrating the biological importance of phages. More importantly, the debate about the influence of HGT in phylogenomics, and especially bacterial phylogenomics, is not covered (eg Gogarten et al 2002 Mol Biol Evol 9:2226, Daubin et al 2003 Science 301:829, Lerat et al 2003 PLoS 1:19, Bapteste et al 2005 BMC Evol Biol 5:33, Galtier 2007 Syst Biol 56:633). I think that the comparison between the bacterial and phage situations would be worth making. The bacterial literature focuses on conflicts between gene trees, whereas your approach relies on the gene-content tree. Finally, the bibliography of gene-content trees could be discussed: is this a well-accepted approach, was it validated is other taxonomic groups?

*Authors' response*: Our discussion of HGT is expanded, with many new references – some of those proposed by the reviewer, as well as a few others. On a more general note, we feel that the debate of HGT in phylogenomics should focus more on the quantitative detection of the actual events than on sweeping metaphors ("promiscuous gene exchange" vs. "formidable barriers", etc.), and hope that our study will contribute to this change of tone, just as many of those newly included references strive to do.

- Any conflict between gene trees and the gene-content tree is interpreted as a HGT event. Given the high turn-over of genes in DNA phages (most POGs are restricted to few phages), isn't it more plausible to interpret a conflict as resulting from several independent acquisitions of the gene (from the same host, or related hosts) in distinct phage lineages?

*Authors' response*: A similar question was raised by Eugene Koonin – see our response to his comments above.

- As far as I understood the modified T-Rex algorithm, every topological conflict between a gene tree and the gene-content tree is interpreted as a HGT event, irrespective of the statistical support for internal branches. I think this will tend to produce an excess of detected HGT in case of poor resolution – two trees can differ by chance in the absence of HGT when the amount of information is low – but the text says more or less the opposite. Is it possible to include only well-supported internal edges in the modified T-rex algorithm? The supplementary method section introduces bootstrap support for HGT, but the main text does not mention it.

*Authors' response*: We were more concerned by underestimation of HGT rates than by their overestimation – therefore the sources of the former were initially discussed in the text in more detail than the sources of the latter. We now expanded the list of "what can go wrong". We agree that this is worth of further investigation, and we are still experimenting with the most appropriate way of using bootstrap analysis for this purpose.

How were irresolutions in the gene-content tree (e.g. a star-like group), if any, dealt with?

*Authors' response*: We did not encounter star-like groups in the gene content tree.

- I think we lack information about the power of the HGT detection approach, because we lack information about the overlap between POGs. Consider a group of phages that would share one or several POGs, but would be involved in no other POG. This group would be well-supported in the gene-content tree, but unfalsifiable through gene-content tree/gene tree comparison, the gene-content subtree being unresolved (this depends on the answer to the previous comment, though). Is there a way to give an idea of how many gene-content groups are corroborated (not just "not contradicted") by gene trees?

*Authors' response*: Since the answer to the previous question is "no well-supported but unresolved subtrees", there remains only the question of the proper "support index", i.e., how many sequence-family trees support each gene-content subtree. We experimented with this index, which can be derived by comparing the number of sequence family trees that give the same topology as the gene-content tree to the number of sequence family trees that show any type of HGTs for a phage genome or for a phage group. The issue here is that the index has to be simultaneously normalized for genome sizes and for group sizes. We have not completed this work yet.

### Reviewer 3: Martijn Huynen, University of Nijmegen

The paper by Glazko et al presents a gene content approach to deriving a phylogeny of the double stranded bacteriophages. As no genes are shared between all double stranded bacteriophages this appears a sensible approach to bacteriophage taxonomy that was introduced by Rohwer and Edwards.

I am not much of a specialist on bacteriophage evolution, and thus my review will focus more on the technical and conceptual issues. In general, gene content approaches can be an alternative to sequence-based trees, although when ample sequences are available that are shared between all species compared, the latter approach includes more information and appears to obtain better trees. With respect to Bacteriophage evolution I wonder whether the authors can include more information about whether this taxonomy is interesting beyond the result itself: are there phylogenetic clusters that e.g. match with host preference or other aspects of lifestyle?

*Authors' response*: There is some correlation between membership in the same group and overlaps in the host range. We now mention this, and we also note that such correlation cannot be explained solely by gene shuffling in viruses that share hosts (see discussion of HGT and tree built after the omission of horizontally transferred genes).

I very much like the quantitative analyses of the authors that relates the number of HGT events to the number of POGs and genomes. Would it be possible to somehow quantify this further into the maximum number of HGT events that could be observed? Or, how dominant is vertical inheritance relative to HGT?

*Authors' response*: In effect, we are already estimating the maximal number of possible HGT events on each gene-content subtree defined by a given sequence-family tree: we do not use any threshold for accepting HGTs, so each of these inferred events is reported. A better way to estimate the HGT rate may be to use a bootstrap scheme (cf. response to Nicolas Galtier's comment). We outlined one such scheme in the supplementary material, but did not use it in this study.

In my opinion the discussion about whether one can derive species trees from genes in the face of HGT is very much confounded by arguments that, indeed, when we search long enough we will likely find that every Orthologous Group has been subject to HGT, but that does not imply that the major mode of inheritance is not a vertical one, and that therefore gene-based phylogenies cannot be derived.

*Authors' response*: We agree.

The remark that the "COG"-algorithm is more sensitive than other methods for deriving orthologous groups because it does not rely on the arbitrary score cut-off, with the reference to the paper that introduced COGs, and that uses the same, theoretical argument, is in my view a bit of a stretch. In absence of golden standards we actually do not know what is the best way of deriving orthologous groups, and whether there is actually one way that works best for all applications. COGs are a sensible approach and they work fine. Please leave it at that.

*Authors' response*: If there is a symmetric best match between two genes in two genomes with the P-value of 0.1 (such a high number may be the consequence of low sequence similarity), and we apply a method of ortholog definition that only examines matches with the P-value of 10^-5 ^or less, such a method would miss such a pair. In contrast, when top-ranking matches are used regardless of the score or P-value, the pair will not be missed. This is our claim, which we think is not controversial. We agree, however, that the extent of gain in orthologs when using ranks is not well-examined. It is our sense that in phages, with lots of short, highly diverged genes, such a gain may be non-trivial, but we agree that a quantitative study is in order. For the time being, we have toned the rhetoric down.

The authors do mention the problem of parallel gene loss leading to convergence in the gene content trees. One way to get around that is to make a tree that only considers the fraction of shared genes (POGs in this case) as a similarity criterion, rather than one that does also include shared "absences". This method has been shown to give "better" trees of bacterial genomes that have undergone parallel loss of genes, and it might be useful to test whether such an approach does lead to a radically different tree in this case.

*Authors' response*: The distance used for building our neighbour-joining tree belongs to the parametric family discussed in ref. 36, which possesses exactly this property (zeroes do not contribute to the distance).

The observation that leaving out likely HGT genes from the tree actually reduces the bootstrap support, is similar to what we have seen for Gene Content trees of cellular species (Dutilh et al., JME 2004) where also removal of likely HGT genes reduced bootstrap support in the tree.

*Authors' response*: Indeed. This reference is now included.

## Supplementary Material

Additional file 1 TablesS1-S5. Click here for file

Additional file 2 **FigureS1**. Gene content tree of dsDNA bacteriophages constructed with MrBayes program. Click here for file

Additional file 3 SupplementaryMethods. Click here for file

Additional file 4 **FigureS2**. Distribution of HGT events in phages and in POGs. Click here for file
