# Supplementary material for: Evolutionary history of bacteriophages with double-stranded DNA genomes
Source: Biol Direct. 2007 Dec 6;2:36. doi: 10.1186/1745-6150-2-36 (PMC2222618; doi:10.1186/1745-6150-2-36)
Supplement: Additional file 1 — TablesS1-S5. [file 1745-6150-2-36-S1.doc]

Table S1. Description of phage groups that have high (above 50%) statistical support in the gene content trees.

| N | Phages included | Group type ^a^ | NCBI taxonomy affiliation | Notes and Comments |
| --- | --- | --- | --- | --- |
| 1 | 315.4,PhiNIH1.1,MM1,A118,phig1e | 3 | Siphoviruses | All infect Bacilli |
| 2 | phi11,phiETA,phi12,phiSLT,77,phiN315,phiPV83,PVL,phi13 | 2 | Siphoviruses | All infect *Staphylococcus aureus*. |
| 3 | BK5-T,P335,bIL309,bIL285,bIL286,TP901-1,Tuc2009,ul36 | 2 | Siphoviruses | All infect *Streptococcus thermophilus*. |
| 4 | 315.3,SM1,phiLC3,r1t | 3 | Siphoviruses | All infect Bacilli |
| 5 | C2,bIL170,sk1 | 2 | Siphoviruses | All infect *Lactococcus lactis*. |
| 6 | 7201,DT1,Sfi19,Sfi21,phiO1205,Sfi11 | 2 | Siphoviruses | All infect *Lactococcus lactis*. |
| 7 | SSV1,SSVRH,SSV2,SSVK1 | 1 | Fuselloviruses | All infect *Sulfolobus* |
| 8 | BIP-1,BPP-1,BMP-1,epsilon15 | 2 | Podoviruses |  |
| 9 | B103,PZA,GA-1,Cp-1,C1,44AHJD,phiP68,P1,Bam35c,PRD1 | 3 | 7 phi29-like podoviruses, 1 myovirus,  2 tectiviruses | phi-29 podoviruses and tectiviruses are morphologically distinct. |
| 10 | Bcep43,Bcep781,Bcep1 | 2 | myoviruses | Infect *Burkholderia cepacia* |
| 11 | lambda,phi4795,933W,VT2-Sa,Stx2I,Stx1,Stx2II | 1 | lambda-like siphoviruses |  |
| 12 | Sf6,ST64T,HK620,P22 | 1 | P22-like podoviruses |  |
| 13 | Che8,Che9d,Che9c,Corndog,CJW1,Omega,TM4,Bxz1,Bxb1,Bxz2,D29,L5,Barnyard,PG1,Rosebush | 3 | 14 siphoviruses,  1 myovirus (Bxz1) | All infect *Mycobacterium* species. In the NJ tree, two subgroups, represented by L5 and Omega are supported with jackknife values of 100% and 45% individually |
| 14 | RB69,T4,44RR28.t,RB49,Aeh1,KVP40,RM378 | 1 | T4-like myoviruses |  |
| 15 | SP6,phiKMV,P60,Gh-1,T3,phiYeO-312,T7,phiA1122 | 1 | T7-like podoviruses |  |
| 16 | ST64B,SfV,phiP27 | 2 | myoviruses | phiP27, SfV, and ST64B are lambda/Mu chimeras, i.e. their head genes are lambda-like, while tail genes are Mu-like.  SfV is unclassified myovirus in the NCBI taxonomy. EM indicates that it belongs to the *Myoviridae* morphology family (Allison et al. 2003). For ST64B, some of its tail genes are inactivated by the insertion of fragments of virulence-related genes, which results in a short-tail pseudo-podovirus morphology. |
| 17 | psiM100,psiM2,PhiCh1 | 3 | 2 siphoviruses,  1 myovirus (PhiCh1) | 3 archaeal phages |
| 18 | L-413C,P2,WPhi,PSP3, 186,phiCTX,K139,HP1,HP2 | 1 | P2-like myoviruses | In the NJ tree, two subgroups, represented by HP1 and P2 are supported with bootstrap value of 100% and 99% individually. |

^a^ Type 1, group includes phages from one ICTV-approved genus; Type 2, group includes phages from an ICTV genus and additional representatives from the same ICTV-approved family; Type 3, group of morphologically diverse or unclassified phages..

Table S2. Putative acts of gene transfer detected by the T-REX algorithm.

|  | Donor | Recipient |
| --- | --- | --- |
| POG11 | D3 | HK022 |
|  | phi3626 | phi-105 |
|  | 315.5 | Rosebush |
|  | SPP1 | phiETA |
| POG12 | Felix01 | PY54 |
| POG22 | Stx1 | Stx2I |
| POG28 | Sfi21 | Sfi11 |
|  | BcepNazgul | phi12 |
| POG29 | phi4795 | HK620 |
| POG30 | PRD1 | Aaphi23 |
|  | Bcep43 | Bcep1 |
| POG33 | phi-105 | phig1e |
| POG34 | lambda | Sf6 |
| POG35 | HK022 | HK620 |
| POG37 | HK022 | P22 |
| POG41 | phBC6A51 | Che8 |
| POG44 | PG1 | Corndog |
| POG71 | Che8 | Bxb1 |
| POG73 | Barnyard | Che9d |
|  | Che8 | Corndog |
|  | TM4 | CJW1 |
| POG86 | epsilon15 | SPP1 |
| POG87 | PG1 | Che9d |
|  | CJW1 | Bxb1 |
| POG99 | phi-105 | Bxz1 |
| POG110 | phiSLT | PVL |
|  | phi13 | phi12 |
| POG115 | phiSLT | PVL |
| POG116 | phiN315 | phi13 |
|  | phiPV83 | phiETA |
| POG123 | phiSLT | phi11 |
| POG124 | phiSLT | phi11 |
| POG126 | phi13 | phi11 |
|  | phiN315 | phiSLT |
|  | 77 | phi12 |
| POG127 | phi12 | 77 |
|  | phiPV83 | phiN315 |
| POG129 | phiSLT | phiPV83 |
|  | phiN315 | PVL |
| POG131 | phiN315 | phiPV83 |
|  | phi12 | phi11 |
| POG132 | 77 | phiPV83 |
| POG143 | phiSLT | phiETA |
| POG144 | SPP1 | SPB |
| POG153 | bIL286 | bIL285 |
| POG154 | Tuc2009 | BK5-T |
| POG157 | Tuc2009 | bIL285 |
| POG160 | BK5-T | Tuc2009 |
|  | bIL286 | ul36 |
| POG161 | r1t | Tuc2009 |
| POG162 | Tuc2009 | bIL286 |
| POG163 | ul36 | P335 |
| POG164 | bIL309 | ul36 |
| POG166 | Tuc2009 | r1t |
| POG167 | Sfi19 | Sfi11 |
| POG168 | BK5-T | bIL285 |
| POG170 | BK5-T | bIL285 |
|  | bIL286 | bIL309 |
| POG172 | ul36 | Tuc2009 |
| POG173 | phiLC3 | TP901-1 |
| POG175 | bIL310 | Lj928 |
| POG177 | P335 | bIL286 |
| POG180 | BK5-T | bIL286 |
| POG181 | A2 | 315.2 |
|  | Sfi19 | DT1 |
|  | bIL286 | P335 |
|  | phiPV83 | phi13 |
| POG182 | DT1 | Sfi21 |
| POG184 | P335 | BK5-T |
|  | 7201 | Sfi19 |
| POG185 | Sfi19 | 7201 |
|  | BK5-T | bIL286 |
| POG186 | bIL286 | bIL309 |
| POG187 | phiO1205 | 7201 |
| POG189 | r1t | ul36 |
|  | TP901-1 | phiLC3 |
| POG195 | Sfi21 | phiO1205 |
| POG198 | bIL312 | MM1 |
| POG203 | 315.5 | 315.1 |
|  | 77 | phi12 |
| POG204 | SPP1 | phiETA |
| POG208 | phi11 | 315.6 |
|  | Tuc2009 | ul36 |
| POG210 | phiETA | SPP1 |
|  | Tuc2009 | TP901-1 |
| POG211 | 315.6 | SPP1 |
|  | ul36 | Tuc2009 |
| POG212 | Tuc2009 | TP901-1 |
| POG213 | Tuc2009 | TP901-1 |
| POG215 | Tuc2009 | ul36 |
| POG218 | HK620 | P22 |
| POG221 | HK97 | HK620 |
| POG222 | Stx2II | VT2-Sa |
| POG223 | P22 | Sf6 |
|  | Stx2I | 933W |
| POG224 | Stx2II | VT2-Sa |
|  | phi4795 | HK97 |
|  | Stx2I | 933W |
| POG226 | HK620 | P22 |
|  | 933W | Stx2I |
| POG228 | Aaphi23 | HK620 |
| POG229 | ST64T | lambda |
|  | Stx2II | HK97 |
| POG230 | lambda | Sf6 |
| POG233 | HK022 | HK620 |
|  | lambda | Sf6 |
| POG241 | Sf6 | HK620 |
| POG242 | Sf6 | HK620 |
| POG243 | Sf6 | HK620 |
| POG247 | P22 | HK620 |
| POG249 | ST64T | Sf6 |
|  | APSE-1 | P22 |
| POG252 | TP901-1 | P335 |
| POG256 | Aaphi23 | P335 |
|  | Sfi19 | Sfi11 |
| POG257 | Lj928 | phig1e |
| POG263 | A118 | EJ-1 |
|  | phiETA | phBC6A51 |
| POG266 | MM1 | phig1e |
| POG271 | MM1 | SM1 |
| POG282 | EJ-1 | MM1 |
|  | phiA1122 | PSA |
|  | phiN315 | phi12 |
| POG284 | Lj928 | phiadh |
|  | 315.4 | 315.2 |
|  | A118 | phBC6A52 |
| POG285 | EJ-1 | MM1 |
| POG291 | 315.6 | 315.2 |
|  | Sfi21 | phiO1205 |
|  | Sfi19 | Sfi11 |
| POG292 | T3 | phiA1122 |
| POG297 | A118 | phiETA |
| POG302 | K139 | phiCTX |
| POG321 | phiCTX | SfV |
| POG324 | phiETA | phiPV83 |
| POG326 | phi11 | phi13 |
| POG328 | phi11 | phiPV83 |
|  | 77 | phiETA |
| POG330 | phiETA | phiPV83 |
|  | phi12 | 77 |
| POG331 | phiETA | phi13 |
| POG332 | phiETA | 77 |
| POG333 | PVL | phiPV83 |
| POG341 | phiA1122 | T7 |
| POG343 | phiA1122 | T7 |
| POG344 | phiA1122 | T7 |
| POG345 | T7 | phiA1122 |
| POG348 | T7 | phiA1122 |
| POG349 | T7 | phiA1122 |
| POG350 | phiA1122 | T3 |
| POG352 | phiA1122 | T7 |
| POG353 | T7 | phiA1122 |
| POG354 | phiA1122 | T7 |
| POG355 | phiA1122 | T7 |
|  | phiKMV | SP6 |
| POG356 | phiA1122 | T7 |
| POG359 | phiA1122 | T7 |
| POG360 | T7 | phiA1122 |
| POG361 | T7 | phiA1122 |
| POG362 | T7 | phiA1122 |
| POG364 | T3 | phiA1122 |
| POG365 | T3 | phiA1122 |
| POG366 | T3 | phiA1122 |
| POG367 | T7 | phiA1122 |
| POG369 | phiA1122 | T7 |
| POG396 | Stx2I | phi4795 |
|  | HK97 | Stx1 |
| POG409 | 933W | Stx2II |
| POG410 | phi4795 | Stx1 |
| POG411 | phi4795 | Stx1 |
| POG412 | phiCTX | D3 |
| POG414 | Xp10 | phi1026b |
|  | phiP27 | ST64B |
| POG428 | VWB | Che9d |
| POG430 | CJW1 | TM4 |
| POG432 | TM4 | KVP40 |
| POG435 | Stx2II | 933W |
| POG439 | Sfi11 | Sfi19 |
|  | EJ-1 | SM1 |
| POG440 | 7201 | DT1 |
|  | Sfi21 | phiO1205 |
|  | Sfi19 | Sfi11 |
| POG441 | 7201 | Sfi19 |
| POG442 | Sfi19 | phiO1205 |
|  | DT1 | 7201 |
| POG443 | SSV1 | SSVK1 |
| POG456 | 44RR28.t | Aeh1 |
| POG467 | Stx2II | 933W |
| POG522 | Stx2I | Stx1 |
| POG526 | 933W | Stx2II |
| POG533 | Stx2I | 933W |
| POG535 | Stx2II | VT2-Sa |
|  | phi4795 | Stx2I |
| POG536 | Stx2I | 933W |
| POG543 | Stx2II | VT2-Sa |
| POG544 | Stx2II | VT2-Sa |
|  | phi4795 | Stx2I |
| POG549 | phi4795 | Stx2I |
| POG551 | Stx1 | VT2-Sa |
| POG553 | 933W | phi4795 |
| POG557 | Stx1 | Stx2I |
| POG558 | VT2-Sa | Stx1 |
| POG562 | Stx2II | 933W |
| POG563 | Stx2I | Stx1 |
| POG570 | PY54 | A2 |
|  | phiPV83 | phi13 |
| POG571 | phi-105 | phi3626 |
| POG589 | Sfi21 | Sfi11 |
| POG599 | Felix01 | Aaphi23 |
| POG606 | Stx2II | 933W |
|  | VT2-Sa | phi4795 |
|  | T3 | phiA1122 |
|  | HK97 | Sf6 |
| POG610 | Bcep781 | Bcep1 |
|  | BPP-1 | BIP-1 |
| POG631 | EJ-1 | Aeh1 |
| POG634 | Bcep43 | Bcep1 |
| POG639 | lambda | Sf6 |
| POG640 | Stx2II | VT2-Sa |
| POG653 | phiPV83 | phi13 |
| POG654 | 315.6 | 315.2 |
| POG655 | 315.5 | 315.4 |
| POG670 | 77 | PVL |
| POG671 | phi13 | 77 |
| POG673 | phiKZ | 44RR28.t |
| POG688 | Che9c | Corndog |
| POG690 | Corndog | Che8 |
| POG691 | Che8 | Corndog |
| POG692 | Che9c | Corndog |
| POG693 | Omega | Che8 |
| POG702 | Che8 | Corndog |
| POG703 | Che9c | Corndog |
| POG716 | Bxz2 | APSE-1 |
| POG722 | Che9c | Corndog |
| POG725 | Rosebush | Barnyard |
| POG732 | 44RR28.t | RB49 |
| POG734 | 44RR28.t | RB49 |
| POG737 | Aeh1 | 44RR28.t |
| POG739 | 44RR28.t | RB49 |
| POG742 | HP1 | K139 |
| POG754 | BMP-1 | BPP-1 |
| POG765 | RB49 | Aeh1 |
| POG772 | RB49 | 44RR28.t |
| POG774 | Aeh1 | RB49 |
| POG775 | 44RR28.t | Aeh1 |
| POG776 | T4 | RB49 |
| POG779 | 44RR28.t | RB49 |
| POG785 | KVP40 | RB49 |
| POG792 | KVP40 | 44RR28.t |
| POG802 | 44RR28.t | RB49 |
| POG805 | 44RR28.t | RB49 |
| POG806 | 44RR28.t | RB49 |
| POG807 | KVP40 | RB49 |
| POG809 | 44RR28.t | Aeh1 |
| POG810 | RB49 | Aeh1 |
| POG815 | Aeh1 | 44RR28.t |
| POG817 | RB49 | KVP40 |
| POG825 | KVP40 | 44RR28.t |
| POG829 | RB69 | Aeh1 |
|  | 44RR28.t | RB49 |
| POG833 | 44RR28.t | RB49 |
| POG836 | 44RR28.t | Aeh1 |
| POG837 | 44RR28.t | RB49 |
| POG842 | Felix01 | KVP40 |
| POG852 | lambda | N15 |
| POG854 | SSVRH | SSV2 |
|  | SSVK1 | SSV1 |
| POG855 | SSVK1 | SSV1 |
| POG860 | SSVRH | SSVK1 |
| POG862 | SSVRH | SSV2 |
| POG863 | SSVRH | SSV2 |
| POG865 | SSV2 | SSVRH |
|  | SSV1 | SSVK1 |
| POG870 | SSVRH | SSV2 |
|  | SSVK1 | SSV1 |
| POG902 | SPP1 | Che9c |
| POG908 | bIL285 | Tuc2009 |
| POG909 | r1t | TP901-1 |
|  | bIL170 | Tuc2009 |
| POG913 | 44RR28.t | RB49 |
| POG925 | T3 | phiA1122 |
| POG927 | Sfi11 | 7201 |
| POG931 | Sfi19 | Sfi11 |
|  | phiadh | A2 |
| POG934 | Aeh1 | 44RR28.t |
| POG937 | P22 | D3 |
| POG952 | RB49 | 44RR28.t |
| POG956 | RB49 | 44RR28.t |
| POG966 | D3 | HK022 |
|  | HK97 | Xp10 |
| POG969 | bIL286 | Sfi21 |
| POG974 | Omega | Che9c |
| POG981 | APSE-1 | SfV |
| POG985 | Aeh1 | 44RR28.t |

Table S3. Phage genomes most frequently donating and receiving genes from other phages. Phage names are followed by the group number in parentheses (“-” indicates that the phage was not included in any of the 18 groups described in the text).

| ***Phages most active as recipients*** | |
| --- | --- |
| *Donor phage* | *Recipient phages* |
| Stx1(11) | HK97(-):1;Stx2I(11):1;VT2-Sa(11):1; phi4795(11):2 |
| Stx2I(11) | 933W(11):1; Stx1(11):2;phi4795(11):2 |
| T7(15) | phiA1122(15):5 |
| TP901-1(3) | Tuc2009(3):3;r1t(3):1; PhiLC3(4):1 |
| Tuc2009(3) | BK5-T(3):1;bIL170(3):1; r1t(3):1;ul36(3):2 |
| phi12(2) | BcepNazgul(-):1; 77(2):2; phi13(2):1; phiN315(2):1 |
| phi13(2) | phi11(2):1; phiN315(2):1;phiPV83(2):3 |
| phiPV83(2) | 77(2):2; phi11(2):1; phiETA(2):1; phiN315(2):1; phiSLT(2):1 |
| 44RR28.t(14) | Aeh1(14):2; KVP40(14):1;RB49 (14):3 |
| Sf6(12) | HK97(-):1;P22(12):1;ST64T(12):1;lambda(11):3 |
| VT2-Sa(11) | Stx1(11):1; Stx2II(11):5 |
| phiETA(2) | A118(1):1;SPP1(-):2; 77(2):1; phiPV83(2):1;phiSLT(2):1 |
| Sfi11(6) | Sfi19(6):5;Sfi21(6):2 |
| 933W(11) | Stx2I(11):4; Stx2II(11):4 |
| Aeh1(14) | 44RR28.t(14):4; EJ-1(-):1; RB49(14):2;RB69(14):1 |
| HK620(12) | Aaphi23(-):1; HK022(-):2; HK97(-):1; P22(12):1; Sf6(12):3; phi4795(11):1 |
| phiA1122(15) | T7(15):5;T3(15):5 |
| RB49(14) | 44RR28.t(14):11;Aeh1(14):1;KVP40(14):2;T4(14):1 |
| ***Phages most active as donors*** | |
| *Donor phage* | *Recipient phages* |
| BK5-T(3) | Tuc2009(3):1;biL285(3):2;biL286(3):2 |
| Sfi21(6) | phiO1205(6):3;Sfi11(6):2 |
| T3(15) | phiA1122(15):5 |
| T7(15) | phiA1122(15):5 |
| 77(2) | PVL(2):1;phi12(2):2;phiETA(2):1;phiPV83(2):1 |
| phiN315(2) | PVL(2):1;phi12(2):1;phi13(2):1; phiPV83(2):1;phiSLT(2):1 |
| phiPV83(2) | phi13(2):1; phiETA(2):1; phiN315(2):1 |
| RB49(14) | 44RR28.t(14):3;Aeh1(14):2;KVP40(14):1 |
| Stx2I(11) | 933W(11):4; Stx1(11):2;phi4795(11):1 |
| phi4795(11) | HK620(12):1;HK97(-):1;Stx1(11):2; Stx2I(11):2 |
| phiSLT(2) | PVL(2):2;phi11(2):2;phiETA(2):1; phiPV83(2):1 |
| phiA1122(15) | T7(15):5;T3(15):1;PSA(-):1 |
| Sfi19(6) | DT1(6):1; phiO1205(6):1;Sfi11(6):5; 7201(6):1 |
| Tuc2009(3) | BK5-T(3):1;TP901-1(3):3;bIL285(3):1; bIL286(3):1;r1t(3):1;ul36(3):2 |
| Stx2II(11) | 933W(11):4; HK97(-):1; VT2-Sa(11):5 |
| 44RR28.t(14) | Aeh1(14):1;RB49 (14):11 |

Table S4. Functions of POGs that have been transferred more than once.

| POG ID | Number of proteins | Number of genomes | Function. |
| --- | --- | --- | --- |
| POG11 | 27 | 27 | putative structural protein |
| POG28 | 12 | 12 |  |
| POG30 | 9 | 6 | putative holin |
| POG73 | 15 | 14 | peptidoglycan-binding domain |
| POG87 | 6 | 6 |  |
| POG110 | 5 | 5 | Putative membrane protein |
| POG116 | 8 | 8 |  |
| POG126 | 9 | 9 |  |
| POG127 | 10 | 9 |  |
| POG129 | 6 | 6 |  |
| POG131 | 8 | 8 | rinB |
| POG160 | 8 | 8 |  |
| POG170 | 10 | 10 |  |
| POG181 | 14 | 14 | putative DNA packaging protein |
| POG184 | 10 | 10 | major structural protein (tail) |
| POG185 | 7 | 7 | putative tail component |
| POG189 | 9 | 7 | putative repressor |
| POG203 | 13 | 13 |  |
| POG208 | 9 | 9 |  |
| POG210 | 8 | 8 | tail protein |
| POG211 | 10 | 10 | major tail protein |
| POG223 | 10 | 10 | regulatory protein cIII |
| POG224 | 9 | 9 | antitermination protein N |
| POG226 | 14 | 14 | cII transcription regulator |
| POG229 | 10 | 10 | NinE protein |
| POG233 | 10 | 9 | holin |
| POG249 | 5 | 5 | DNA transfer protein |
| POG256 | 18 | 17 | NTP-binding protein |
| POG263 | 7 | 7 | putative scaffold protein |
| POG282 | 22 | 22 |  |
| POG284 | 16 | 16 | ArpU family transcription regulator |
| POG291 | 15 | 15 |  |
| POG328 | 6 | 6 |  |
| POG330 | 5 | 5 |  |
| POG355 | 12 | 12 | head-to-tail joining protein |
| POG396 | 13 | 13 | dam DNA adenine methylase |
| POG414 | 6 | 6 |  |
| POG439 | 9 | 9 |  |
| POG440 | 6 | 6 |  |
| POG442 | 6 | 6 |  |
| POG535 | 7 | 7 |  |
| POG544 | 7 | 7 | ea10, putative ssDNA binding protein |
| POG570 | 6 | 6 | putative head-tail joining protein |
| POG606 | 23 | 23 | endopeptidase Rz |
| POG610 | 10 | 10 |  |
| POG829 | 6 | 6 | gp31.1 conserved hypothetical protein |
| POG854 | 4 | 4 |  |
| POG865 | 4 | 4 |  |
| POG870 | 4 | 4 |  |
| POG909 | 6 | 6 | neck passage structural protein |
| POG931 | 11 | 11 | putative SSB |
| POG966 | 5 | 5 | structural component |

Table S5. Statistical support of 18 groups with and without horizontally transferred genes.

|  | Group size | Group type ^a^ | Group support, % in jackknife test before/after exclusion of POGs that have been transferred at least once | Loss of genomes in a clade after excluding horizontally transferred genes |
| --- | --- | --- | --- | --- |
| 1 | 5 | 3 | 63 / 53 |  |
| 2 | 9 | 2 | 95 / 9 | -2 |
| 3 | 8 | 2 | 35 / 17 | -1 |
| 4 | 4 | 3 | 72 / 77 |  |
| 5 | 3 | 2 | 96 / 87 |  |
| 6 | 6 | 2 | 54 / 63 |  |
| 7 | 4 | 1 | 100 / 100 |  |
| 8 | 4 | 2 | 77 / 61 |  |
| 9 | 10 | 3 | 58 / 49 |  |
| 10 | 3 | 2 | 100 / 100 |  |
| 11 | 7 | 1 | 30 / 95 | -2 |
| 12 | 4 | 1 | 100 / 53 |  |
| 13 | 15 | 3 | 51 / 27 | -2 |
| 14 | 7 | 1 | 96 / 93 |  |
| 15 | 8 | 1 | 46 / 8 |  |
| 16 | 3 | 2 | 87 / 75 |  |
| 17 | 3 | 3 | 85 / 83 |  |
| 18 | 9 | 1 | 94 / 92 |  |

^a^ Type 1, group includes phages from one ICTV-approved genus; Type 2, group includes phages from an ICTV genus and additional representatives from the same ICTV-approved family; Type 3, group of morphologically diverse or unclassified phages..
