# Supplementary material for: Evolutionary history of bacteriophages with double-stranded DNA genomes
Source: Biol Direct. 2007 Dec 6;2:36. doi: 10.1186/1745-6150-2-36 (PMC2222618; doi:10.1186/1745-6150-2-36)
Supplement: Additional file 2 — FigureS1. Gene content tree of dsDNA bacteriophages constructed with MrBayes program. [file 1745-6150-2-36-S2.pdf]

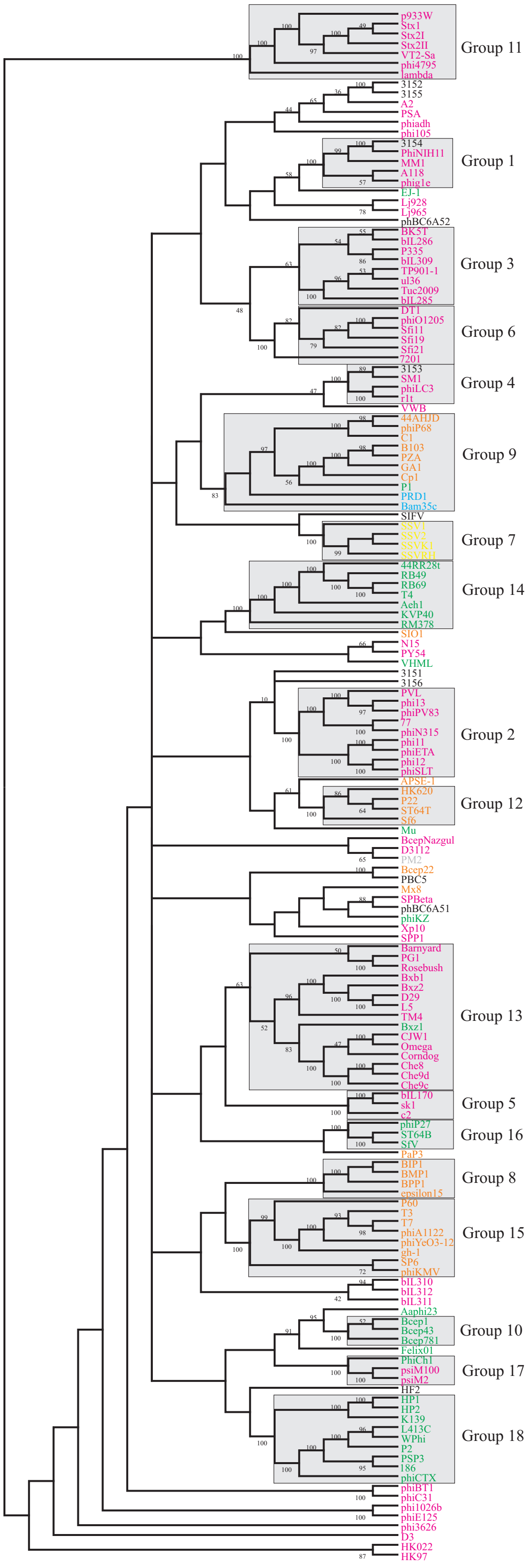

Figure S1. Bacteriophages phylogeny obtained using Bayesian inference. Phages names are colored according to ICTV classification: family *Siphoviridae* is in magenta, *Podoviridae* is in orange, *Myoviridae* is in green, *Fuselloviridae* is in yellow, and *Tectiviridae* is in blue. The single member of *Corticoviridae* family (phage PM2) is in gray. Support values are posterior probabilities generated by MrBayes.
