# Supplementary material for: Evolutionary history of bacteriophages with double-stranded DNA genomes
Source: Biol Direct. 2007 Dec 6;2:36. doi: 10.1186/1745-6150-2-36 (PMC2222618; doi:10.1186/1745-6150-2-36)
Supplement: Additional file 3 — SupplementaryMethods. [file 1745-6150-2-36-S3.doc]

**Additional Materials and Methods**

**Test for a presence of evolutionary signal in data matrix.** Tree inference from ***X***={*x_ij_*}, i.e., the original data matrix of presence/absence of POGs in phage genomes (i=1,…,*n*, j=1,…,*m*; *n*=803, *m*=158), is valid only if the hypothesis “the distribution of POGs among phages reflects mostly the vertical inheritance”, or, in other words “characters distribution in ***X*** is strictly non-random”, holds true. Assume that a presence (1) and absence (0) of a given POG_i_ among the pool of phages is a random variable with probability of success (*p_i_*: *x_ij_*=1) equal to the frequency of 1’s in i^th^ raw of ***X*** ($p_{i}=\sum_{j=1}^{m} x_{ij}/m$), and probability of failure (0) equal to *q_i_*=1-*p_i_*. Then the i^th^ raw of ***X*** can be modeled as a series (of length *m*) of Bernoulli trials. By repeating these trials for every row in data matrix X (*i*=1,…,n) we construct new matrix, ***X^k^*** with row sums equal to those of ***X***, but with elements *x_ij_*^k^ (1’s and 0’s) randomly distributed among the pool of phages (we take here k=1,…,100). These series of matrices correspond to hypotheses that POGs are randomly distributed among phages. We have inferred “semi-random” trees from ***X^k^*** (k=1,…,100) and calculated the probability to obtain a semi-random tree with the average clade support better than the average clade support for the original tree. The neighbor-joining semi-random trees were inferred based on the intergenomic distances $d_{A}\infty$ using the NEIGHBOR program of the PHYLIP package. The statistical support for internal nodes was obtained with the delete-jackknife method and averaged for every semi-random tree over all internal nodes. The distribution of the average clade support per semi-random tree is narrow (mean is equal to12.84, standard deviation is equal to 0.23). The average support for NJ tree inferred from the original data matrix is 53.2. The probability to obtain a semi-random tree with the average clade support better than the average clade support for the original tree is zero.

**Detection of horizontal gene transfers: earlier approaches and the new version of the T-Rex algorithm**

Several algorithmic approaches for inferring reticulation events have been proposed [1-6]. Hallett and Lagergren [6] first presented the exact algorithm for identifying minimum number of horizontal gene transfer events necessary to transform a given species tree into a given gene tree. This algorithm has an exponential time complexity with respect to the number of transfers. Legendre and Makarenkov [7] proposed an efficient reticulogram-building algorithm that produces a reticulate phylogeny by gradually improving upon the initial solution provided by a phylogenetic tree model. Bryant and Moulton [8] introduced a network-inferring method, NeighborNet, allowing the reconstruction of planar phylogenetic networks. The main challenge for all these approaches that any pattern in the data that is suggestive of reticulation may also be due to other factors, such as inherent homoplasy, sampling problems, or inadequate data model [9, 10]. In an ultimate artifact, some of network reconstruction programs operating on a real-life distance matrix may even reticulate a true tree (see for instance ref. 11, and G.Glazko, unpublished observations), and, therefore, a series of external constraints is needed. This section presents the new optimized version of the T-Rex HGT detection algorithm (see refs. [3, 12] for the previous versions).

***Data preprocessing*.** A protein sequence family tree *T_sf_* is a tree inferred from alignment of protein sequences that belong to a POG. This tree has *n* leaves that are labeled by the set of *n* bacteriophages, where *n* is always considerably less than the total number of phages included in the analysis (the average numbers of genes in 450 POGs that contain 4 or more species is 6). We also reduce the 158-taxa gene-content tree, such as the one given in Figure 1 of the main text or Figure S1, to a smaller tree *T_gc_*, containing only the leaves labeled by the set of the same *n* phages, by removing from it 158-*n* lineages corresponding to the organisms missing from the sequence family tree. Both gene-content and sequence family trees are then rooted by midpoint. The algorithm can process the unrooted trees, but rooting allows us to take into account the timing constraints. The transfers within the same lineage as well as double-crossing transfers are not considered - see [6, 12-14] for more detail. If there exist identical sub-trees with two or more leaves belonging to both *T_gc_* and *T_sf_*, we reduce the size of the problem by contracting these sub-trees, replacing them with the same auxiliary node in both *T_gc_* and *T_sf_* , and preserving this replacement throughout the computation (i.e., assuming that the branches of these sub-trees will not be involved in the HGT operations).

***HGT detection based on SPR operations***. All possible directed transformations consisting of standard Sub-tree Pruning and Regrafting (SPR) operations, are evaluated in a way that the value of a selected optimization criterion (in our case, Robinson-Foulds distance, see below) between the transformed species tree and the gene tree is computed. A SPR operation can be defined as follows. First, we select a sub-tree of the given tree. Then, we detach the selected sub-tree and regraft it onto another branch of the remaining tree in such a way that a new tree is formed. The SPR providing the minimum of the selected criterion between the transformed species tree and the gene tree is retained as a solution. Note that the problem of finding the minimum number of SPR operations necessary to transform one tree into another (known as “Sub-tree Transfer Problem”) has been shown to be NP-hard but approximable to within a factor of 3 [15].

***Iterations 1 … k***

1. Test all possible SPR operations (i.e, HGTs) between pairs of branches in the gene-content tree *T_k_*_-1_ (*T*_0_ *= T_gc_* at iteration 1; starting from Iteration 2, we consider the transformed gene-content tree) except the transfers between adjacent branches and those violating the timing (see [6, 12-14]) and sub-tree constraints (see Figure below). The sub-tree constraint discussed in ref. 3 can be formulated in our case as follows. Consider the gene transfer between two phages, i.e., a reticulation in a gene-content tree *T_k_*_-1_ going from branch (phage lineage) *b* to branch *a* and transforming *T_k_*_-1_ into the tree *T_k_* (Fig. A, next page). The following constraint can be postulated: to allow the transfer between the branches (*z*,*w*) and (*x*,*y*) of the gene-content tree *T_k_*_-1_, the cluster combining the sub-trees rooted by the vertices *y* and *w* should be present in the sequence family tree *T_sf_*. Such a constraint enables us, first, to resolve the topological conflicts between *T_gc_* and *T_sf_* that are due to the transfers between single species and, then, to identify the transfers that have occurred in the deep phylogeny. The usage of this constraint allows the method to follow the order that is opposite to the order of HGT and infer first the most recent HGTs, which are easier to detect.

Figure A. Sub-tree constraint: the transfer between the branches (*z*,*w*) and (*x*,*y*) of the species’ gene-content tree *T_k_*_-1_ can be allowed if and only if the sub-tree rooted by *b* and showing the identical topology is present in the sequence family tree. A single branch is depicted by a plain line and a path is depicted by a wavy line.

**B.** If no such transfer exists, relax the sub-tree constraint. In practice, this was observed only in 2% of cases.

**C.** Select the SPR operation (i.e., HGT) that minimizes the Robinson and Foulds topological distance [14] between the transformed gene-content tree *T_k_* and the sequence family tree *T’*. The Robinson-Foulds metric is commonly used to compare the topologies of phylogenetic trees [15]. This distance is equal to the minimum number of elementary operations (i.e., merging and splitting nodes) necessary to transform one tree into the other. As shown in Robinson and Foulds [16], this distance is also the number of bipartitions or Buneman's splits [17] belonging to exactly one of the two trees.

**D.** Similarly to the data preprocessing step, reduce the size of the problem by contracting the newly-formed sub-tree in the transformed gene-content tree *T_k_* and the sequence family tree *T_sf_*. Such a reduction minimizes the number of HGTs and substantially reduces the algorithmic time complexity.

**E.** In the list of the obtained HGTs, search for and eliminate the idle transfers using a dynamic programming procedure. An idle transfer is the transfer whose removal does not change the topology of *T_k_*. In fact, this novel step is recommended for any HGT-inferring algorithm in order to optimize the obtained solution.

**F.** If the Robinson and Foulds distance between the transformed gene-content tree *T_k_* and the sequence family tree *T_sf_* equals zero or if no more HGTs can be generated due to the violation of timing constraints, stop the procedure. Otherwise, go to *Step A*.

***Bootstrap support for horizontal gene transfers*.**

We designed a procedure for computing the bootstrap score of a specific gene transfer identified by our algorithm. The aligned sites in sequences used to build the protein family trees were drawn with replacement in order to create a series of pseudo-replicates. For each HGT branch, we computed the fraction of times that it was obtained with the fixed reduced gene-content and sequence family trees inferred from the sets of pseudo-replicates. Replicated sequence family trees, as well as original sequence family trees, were rooted by midpoint. When the difference in the midpoint locations of the original family tree and that inferred from a replicated sequence family dataset led to the creation of an extra HGT, this HGT was ignored.

Bootstrap analysis can be used to place confidence intervals on internal branches of phylogenetic trees. It involves sampling of original data, with replacement, to create a series of pseudo-samples. We designed the following strategy to evaluate the reliability of horizontal gene transfers. The aligned sites in sequences used to build the protein family trees were drawn with replacement in order to create a series of pseudo-replicates. Thus, for all HGT branches, we verified if they were also present in the transfer scenario found using as input the reduced gene-content tree and the sequence family tree inferred from a set of pseudo-replicates. To compute the bootstrap support of a specific transfer branch, we estimated the ratio of sequence family phylogenies derived from the resampled data and containing the HGT branch in question. Replicated sequence family trees, as well as original sequence family trees, were rooted by midpoint. When the difference in the midpoint locations of the original family tree and that inferred from a replicated sequence family dataset led to the creation of an extra HGT, this HGT was ignored. The option for computing HGT bootstrap scores was included in the new version of the T-Rex package available at: http://www.trex.uqam.ca.

**Summary of the new features in the current version of T-REX**

1. Reduction of the original complete gene-content tree into a smaller tree by removing all the lineages for the species absent in the given sequence family tree.

2. Usage of the sub-tree constraint in addition to the previously proposed timing constraints.

3. Speed-up of the algorithm by iterative removal of identical sub-trees in both gene-content and sequence family trees.

4. Elimination of idle HGTs.

5. Bootstrap analysis of the inferred HGTs.

References to Supplementary Methods

1. B. Moret *et al.*, *IEEE/ACM Trans Comput Biol Bioinform* **1**, 13 (2004).

2. L. Nakhleh, D. Ruths, H. Innan, in *Meta-analysis and combining information in genetics.* R. Guerra, D. Allison, Eds. (2005) pp. 1-27.

3. V. Makarenkov, A. Boc, C. F. Delwiche, A. B. Diallo, H. Philippe, in *Data Science and Classification* V. Batagelj, H.-H. Bock, A. Ferligoj, Z. A., Eds. (Springer Verlag, 2006) pp. 341-349.

4. H. J. Bandelt, A. W. Dress, *Mol Phylogenet Evol* **1**, 242 (1992).

5. D. H. Huson, *Bioinformatics* **14**, 68 (1998).

6. M. Hallett, J. Lagergren, paper presented at the Annual Conference on Research in Computational Molecular Biology Montreal, Quebec, Canada 2001.

7. P. Legendre, V. Makarenkov, *Syst Biol* **51**, 199 (2002).

8. D. Bryant, V. Moulton, *Mol Biol Evol* **21**, 255 (2004).

9. P. Legendre, *Journal of Classification* **17**, 153 (2000).

10. D. H. Huson, D. Bryant, *Mol Biol Evol* **23**, 254 (2006).

11. V. Makarenkov, P. Legendre, *J Comput Biol* **11**, 195 (2004).

12. A. Boc, V. Makarenkov, in *Algorithms in Bioinformatics* G. Benson, R. Page, Eds. (Springer Verlag, Budapest 2003) pp. 190-201.

13. W. P. Maddison, *Syst. Biology* **43**, 523 (1997).

14. R. D. M. Page, M. A. Charleston, *Trends Ecol. Evol.* **13**, 356 (1998).

15. J. Hein, T. Jiang, L. Wang, K. Zhang, *Discr. Appl. Math* **71**, 153 (1996).

16. D. R. Robinson, L. R. Foulds, *Math. Biosciences* **53**, 131 (1981).

18. P. Buneman, in *Mathematics in the Archaeological and Historical Sciences* F. R. Hodson, D. G. Kendall, P. Tautu, Eds. (Edinburgh University Press, Edinburgh, 1971) pp. 387-395.
