# Supplementary material for: Evolutionary history of bacteriophages with double-stranded DNA genomes
Source: Biol Direct. 2007 Dec 6;2:36. doi: 10.1186/1745-6150-2-36 (PMC2222618; doi:10.1186/1745-6150-2-36)
Supplement: Additional file 4 — FigureS2. Distribution of HGT events in phages and in POGs. [file 1745-6150-2-36-S4.pdf]

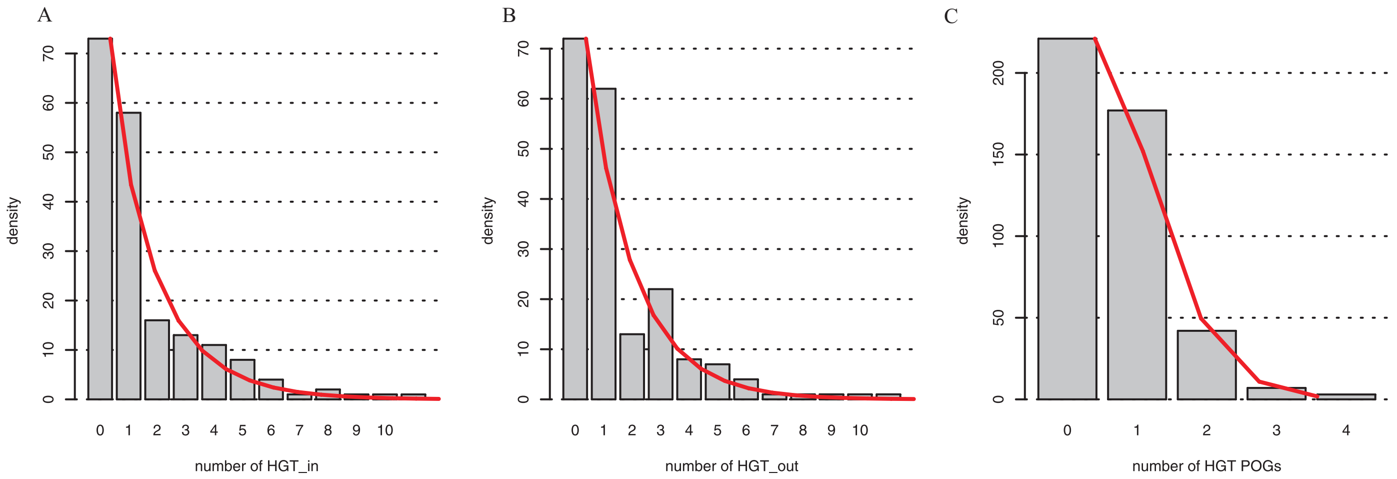

Figure S2. HGT events in phage genomes and in POGs. Phage genomes: A) Distribution of phages by the number of gene gains (HGT-in events). B) Distribution of phages by the number of gene donations (HGT-out events). POGs: C) Distribution of POGs by the number of HGT events detected in each POG. Red lines show fit to negative binomial distribution.
